# Comparison of radiobiological parameters for ^90^Y radionuclide therapy (RNT) and external beam radiotherapy (EBRT) in vitro

**DOI:** 10.1186/s40658-018-0217-8

**Published:** 2018-09-03

**Authors:** Yaser H. Gholami, Kathy P. Willowson, Nicholas J. Forwood, Rozelle Harvie, Nicholas Hardcastle, Regina Bromley, HyunJu Ryu, Samuel Yuen, Viive M. Howell, Zdenka Kuncic, Dale L. Bailey

**Affiliations:** 1 University of Sydney, School of Physics, Sydney, Australia; 2 University of Sydney, Discipline of Medical Radiation Science, Sydney, Australia; 3 Royal North Shore Hospital (RNSH), Department of Nuclear Medicine, Sydney, Australia; 4 Bill Walsh Translational Cancer Research Laboratory, The Kolling Institute, Northern Sydney Local Health District, Sydney, Australia; 5 Royal North Shore Hospital (RNSH), Department of Radiation Oncology, Sydney, Australia; 6 The University of Sydney Northern Clinical School, Faculty of Medicine and Health, The University of Sydney, Sydney, Australia

**Keywords:** Radionuclide therapy (RNT), External beam radiotherapy (EBRT), Yttrium-90

## Abstract

**Background:**

Dose rate variation is a critical factor affecting radionuclide therapy (RNT) efficacy. Relatively few studies to date have investigated the dose rate effect in RNT. Therefore, the aim of this study was to benchmark ^90^Y RNT (at different dose rates) against external beam radiotherapy (EBRT) in vitro and compare cell kill responses between the two irradiation processes.

**Results:**

Three human colorectal carcinoma (CRC) cell lines (HT29, HCT116, SW48) were exposed to ^90^Y doses in the ranges 1–10.4 and 6.2–62.3 Gy with initial dose rates of 0.013–0.13 Gy/hr (low dose rate, LDR) and 0.077–0.77 Gy/hr (high dose rate, HDR), respectively. Results were compared to a 6-MV photon beam doses in the range from 1–9 Gy with constant dose rate of 277 Gy/hr. The cell survival parameters from the linear quadratic (LQ) model were determined. Additionally, Monte Carlo simulations were performed to calculate the average dose, dose rate and the number of hits in the cell nucleus.

For the HT29 cell line, which was the most radioresistant, the *α*/*β* ratio was found to be ≈ 31 for HDR–^90^Y and ≈ 3.5 for EBRT. LDR–^90^Y resulting in insignificant cell death compared to HDR–^90^Y and EBRT. Simulation results also showed for LDR–^90^Y, for doses ≲ 3 Gy, the average number of hits per cell nucleus is ≲ 2 indicating insufficiently delivered lethal dose. For ^90^Y doses $\gtrsim $ 3 Gy the number of hits per nucleus decreases rapidly and falls below ≈ 2 after ≈ 5 days of incubation time. Therefore, our results demonstrate that LDR–^90^Y is radiobiologically less effective than EBRT. However, HDR–^90^Y at ≈ 56 Gy was found to be radiobiologically as effective as acute ≈ 8 Gy EBRT.

**Conclusion:**

These results demonstrate that the efficacy of RNT is dependent on the initial dose rate at which radiation is delivered. Therefore, for a relatively long half-life radionuclide such as ^90^Y, a higher initial activity is required to achieve an outcome as effective as EBRT.

## Background

Radionuclide therapy (RNT) is a fast-evolving and promising modality of treating cancer [1]. Targeted RNT, peptide receptor RNT and selective internal RNT are the most common nuclear medicine cancer therapy procedures for treating different types of cancer metastases [2–5]. With current radiopharmaceutical capabilities, different therapeutic radionuclides such as ^223^Ra, ^213^Bi, ^67^Cu,^32^P, ^177^Lu and ^90^Y can be delivered via microparticles, nanoparticles or small peptide molecules [6].

Current clinical therapeutic radionuclides can be classified based on the nature of their radiation properties such as linear energy transfer (LET, which is defined as the energy deposition per unit length travelled); 50–230 keV/ *μ*m for alpha particle emitters, 0.2 keV/ *μ*m for beta emitters and 4–26 keV/ *μ*m for Auger electron emitters [7, 8]. Radionuclides such as ^223^Ra (*α*),^177^Lu (*β*) and ^67^Cu (*β*) which emit particles with a short range (relatively high LET) are used in Targeted RNT [3, 6]. Due to the short-range particles emitted by these radionuclides, they need to be either attached to the surface of the tumour cell or taken up by the cell to deliver a lethal dose [9]. Alternatively, long-range radionuclide emitters can be labelled with nano- or micro-particles for SIRT [6].

Another important property of therapeutic radionuclides is their physical half-life which determines the dose rate at which RNT is delivered along with its initial activity. For a given initial activity, a radionuclide with shorter half-life delivers dose at higher rate compared to longer half-life radionuclides. The effect of dose rate in RNT and external beam radiotherapy (EBRT) dose response can be classified into low and high dose rates (respectively LDR and HDR) [10, 11]. It is also well known that HDR radiation is biologically more effective than the same dose delivered with LDR [10]. In RNT, as the dose rate decreases exponentially, the kinetics of DNA double-strand break (DSB) induction, repair and misrepair may play a more important role in therapeutic outcome than in EBRT. Although several in vitro studies have reported on EBRT dose rate effects [12, 13], relatively few studies to date have investigated dose rate effects in RNT [14–18]. Consequently, the biological principles of RNT are less well developed.

The aim of this study was to establish a platform for experimental and theoretical ^90^Y RNT in vitro studies and investigate the differences in delivering EBRT and ^90^Y RNT (both HDR and LDR). Additionally, this study aimed to compare cell kill mechanisms between the two irradiation processes. To do this, we investigate ^90^Y RNT, which is a common treatment for primary and metastasised liver cancer that initiates from colorectal cancer.

## Methods

### Cell culture

As this study involved the measurement of radiation survival for a high number of samples, the MTS assay was used instead of the standard clonogenic assay. The human CRC cell lines HT29, HCT116 and SW480 were cultured in RPMI (Roswell Park Memorial Institute) medium supplemented with 10% fetal bovine serum (FBS) and incubated at 37 ^∘^C in a humidified atmosphere containing 5% CO_2_. The cells were assessed by MTS (3-(4,5-dimethylthiazol-2-yl)-5-(3-carboxymethoxyphenyl)-2-(4-sulfophenyl)-2H-tetrazolium) cell viability assay which is a colorimetric quantification of viable cells. The assay is based on the reaction of MTS tetrazolium compound by viable cells to generate a coloured formazan product that is soluble in cell culture media. Since the cell culture medium needs to be changed after 8 days, the maximum incubation time was 8 days. However, ^90^Y requires ≈ 15 days to deliver 98 *%* of the targeted dose. The initial specific activity of ^90^Y was calculated to deliver a target dose in 8 days. All experiments were repeated twice to assess consistency.

### External beam radiotherapy (EBRT)

**Fig. 1:**
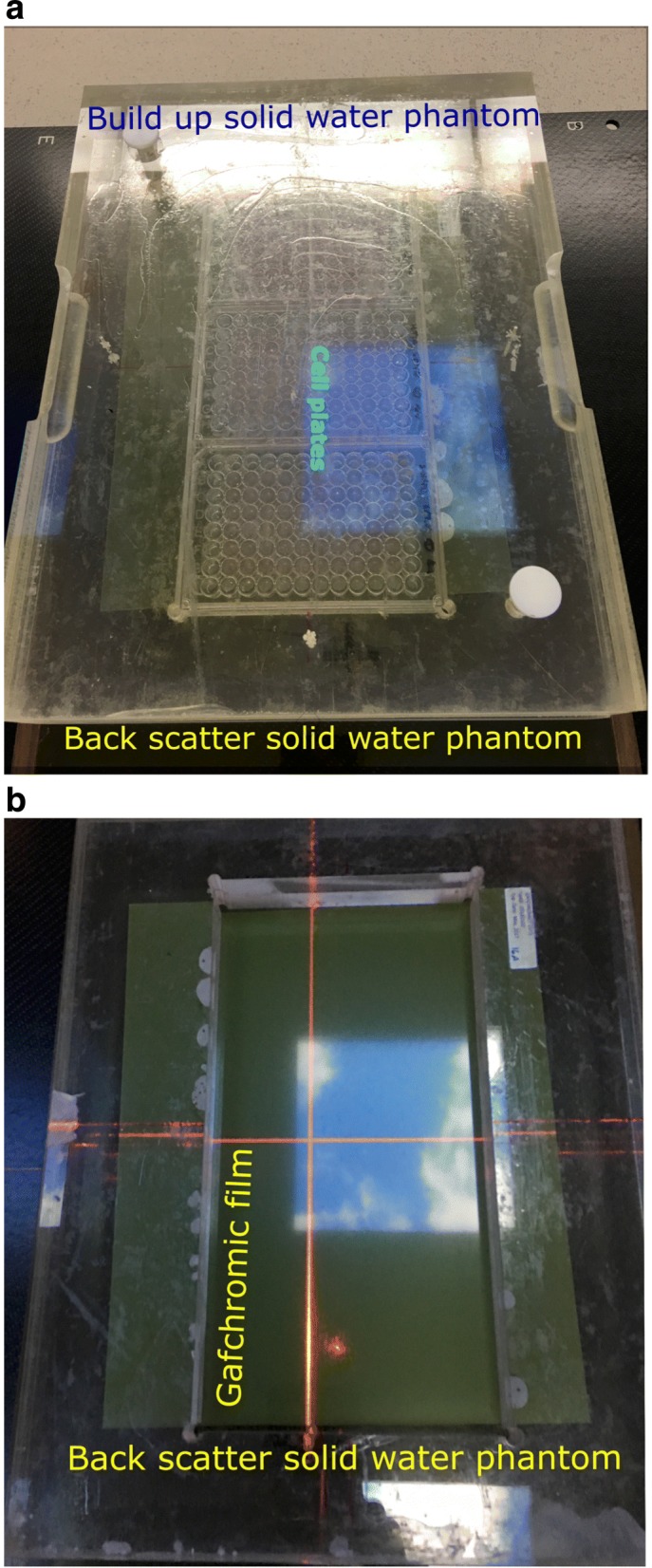
Experimental set-up for irradiating the cells with a 6 MV linac photon beam: **a** cell plates between two solid water phantoms and **b** position of the Gafchromic film

Each cell line was cultured in a 96-well plate and allowed 4 h incubation before irradiation. Additionally, a control cell plate for each cell line was prepared. All of the cell cultures were irradiated with a 6 MV flattening filter-free (FFF) linear accelerator (linac) photon beam with a single dose in the range of ≈ 1–9 Gy (Varian, CAUS). All the cell plates were irradiated at a dose rate of ≈ 277 Gy/hr, at 100 cm source–to–surface distance (SSD), with back-scatter and buildup solid water (see Fig. 1a). A 1-Gy dose gradient was applied across each plate so each cell culture in the well (i.e. each column) received a dose with 1 Gy increments. Also Gafchromic film was placed under the plates to measure the dose in each well (see Fig. 1b). All irradiated cell plates were immediately returned to the incubator for 8 days incubation. The EBRT experiment was repeated three times.

### ^90^Y Irradiation

**Fig. 2:**
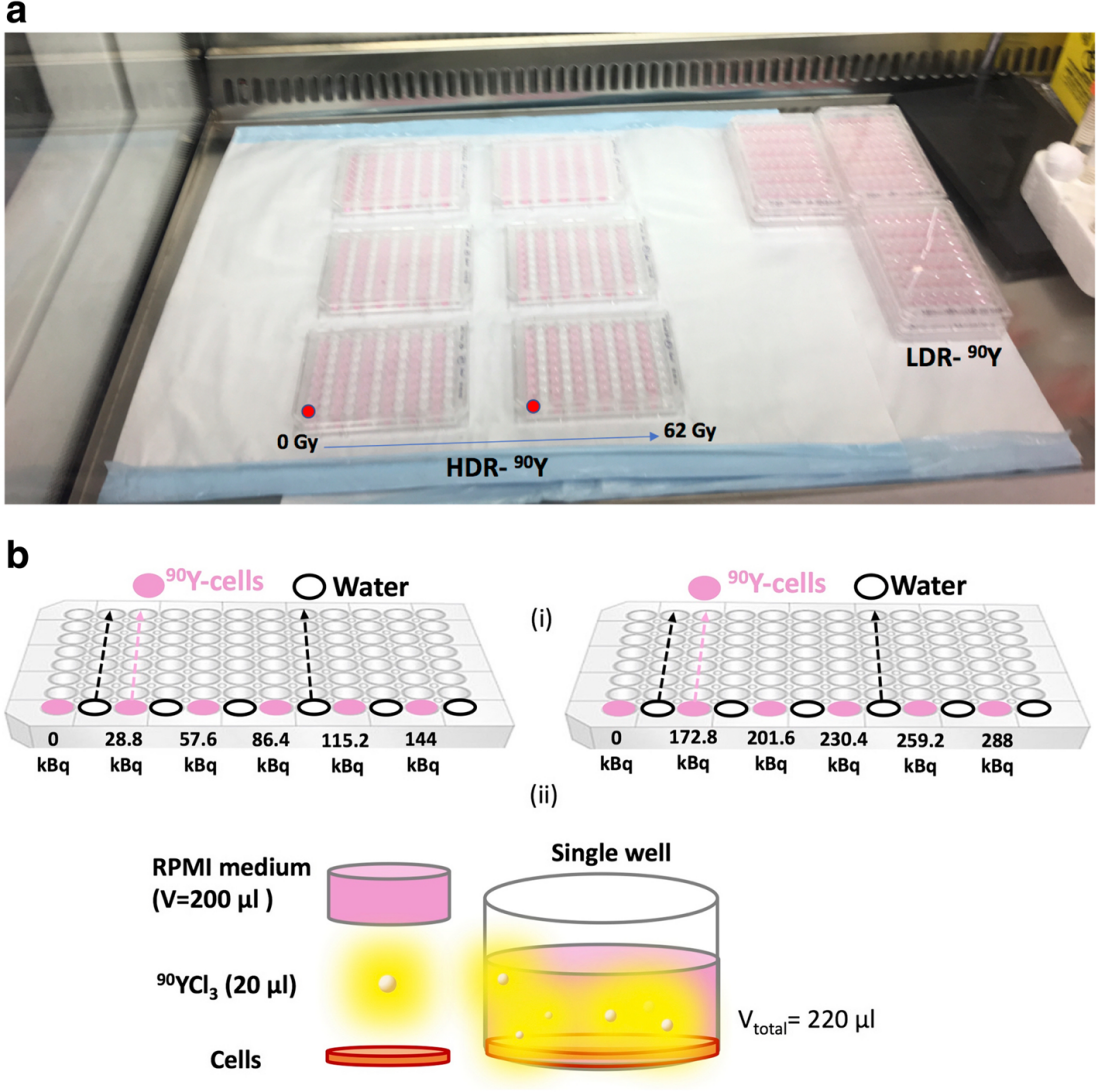
Experimental set-up for ^90^Y. **a** Two sets of ^90^YCl_3_ (HDR, LDR) activity concentrations were added to the cell plates. Each cell line was plated into two plates with range of 0–62 Gy. The red markers show row of wells that contains the cell without activity. **b** Schematic diagram illustrating (i) the distribution of cells and ^90^Y source in two 96-well plates for HDR–^90^Y set (similar set-up was prepared for LDR–^90^Y set), (ii) demonstrating a single-well containing the ^90^YCl_3_ and cells in RPMI cell medium

^90^Y is a beta emitting radionuclide with a physical half-life of ≈  2.7 days. The mean and maximum energy of the emitted betas are 0.933 and 2.280 MeV, respectively, corresponding to an average and maximum range of 4 and 11.3 mm in water [19, 20]. As mentioned above, each cell line was cultured in a 96-well plate and allowed 4 h incubation before adding the ^90^YCl_3_ solution. Additionally, a control cell plate for each cell line was prepared. For each cell line, two identical sets of cell cultures were prepared for irradiation with a uniformly mixed solution of ^90^YCl_3_ in varying concentrations (in 10 dilutions): (1) ^90^YCl_3_ solutions with LDR specific activity of 0.24–2.4 MBq/ml (corresponding to total dose of ≈ 1–10 Gy); and (2) relatively HDR-specific activity of 1.44–14.4 MBq/ml (corresponding to total dose of ≈ 6–62 Gy). The pH of prepared solutions were also measured to be ≈ 7. A volume of 20 *μ*l from each ^90^Y dilution was added to each well (see Fig. 2). Matlab was used to calculate the dose rate according to

1 $$\begin{array}{*{20}l} \dot{D}(t) = \frac{A(t)}{m} = \dot{D}_{0}\,e^{-\lambda\,t} \end{array} $$

where $\dot {D}_{0}$ is the initial dose rate, *λ* is the decay constant, *m* is the target mass, *T* is the total irradiation time (8 days) and *A*(*t*) is activity as a function of time representing the number of beta particles (i.e. number of decays) emitted per second with energies randomly selected from the ^90^Y spectrum. The corresponding total doses were calculated according to

2 $$\begin{array}{@{}rcl@{}} D = \int_{0}^{T} \dot{D}(t)\,dt\, = \dot{D}_{0}\,\int_{0}^{T} e^{-\lambda\,t}\, dt= \frac{\dot{D}_{0}}{\lambda}\,\bigg(1-e^{-\lambda\,T}\bigg) \end{array} $$

for varying ^90^Y concentrations using the full beta energy spectrum of ^90^Y. The physical ^90^Y absorbed doses were calculated to be ≈ 1–10 Gy for LDR and ≈ 6–62 Gy for HDR. Results were also validated by OLINDA [21] and GATE [19] calculations.

### Monte Carlo simulation

A Monte Carlo model was developed in the GATE toolkit [19] to calculate the average dose to the cell for varying ^90^Y concentrations. GATE is a Monte Carlo-based platform developed by the international OpenGATE collaboration for radionuclide imaging and dosimetry applications [19]. All simulations were performed using GATE version 7.1 [19], which uses GEANT4 10.1.p01 [20]. The low-energy electromagnetic physics package [22] of GEANT4, which describes electron, photon, and light ion interactions over an energy range of 250–1 GeV, was used for all simulations.

The energy spectrum of emitted beta particles was obtained using GEANT4 [20, 23] and imported into GATE. The stochastic processes of beta emission and hit distribution, as well as associated energy and dose deposition, were simulated using Monte Carlo radiation transport within GEANT4. The total number of beta particles emitted in a single-well plate was determined using

3 $$  \widetilde{A} = A_{0}\,\int_{0}^{T} e^{-\lambda\,t} dt  $$

where $\widetilde {A}$ represents the total number of nuclear disintegrations (i.e. cumulative activity) and *A*_0_ is the initial activity.

#### Geometry set-up

**Fig. 3:**
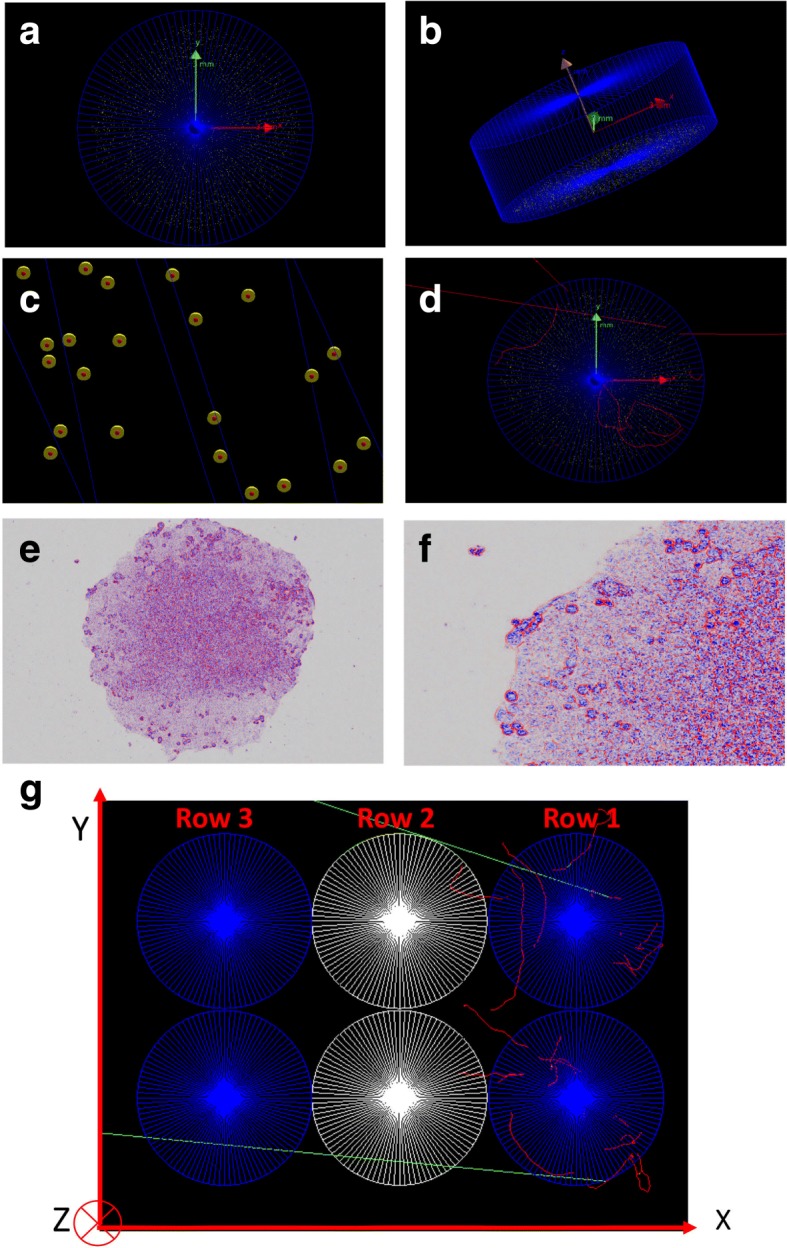
Geometry set-up in the GATE simulations: **a**, **b** 5000 non-overlapping spherical cells randomly distributed in a cylindrical well (axial and cross-sectional views); **c** yellow and red spheres representing the cytoplasm and nucleus respectively; **d** beta particle tracks (red) in the well; **e** microspcopy image of a cell culture configuration in a single well; **f** zoom-in of image in (**e**); **g** six adjacent wells in three rows: row 1: active rows with each well containing 288 kBq ^90^YCl_3_ in 200 *μ*l cell culture medium, row 2: wells with 200 *μ*l of water, and row 3: with two well only containing 200 *μ*l cell culture medium. The red and green tracks represent emitted beta particles and scattered photons (from the atomic de-excitations), respectively

A monolayer of 5000 non-overlapping spherical “cells” was randomly placed at the bottom of a 200 *μ*l cylindrical well (Fig. 3a–c) to mimic the actual cell culture geometry (see Fig. 3e, f). The average diameter of the cell lines used in this study is approximately 15 *μ*m [24]. Therefore, cells were modelled as spheres with diameter 15 *μ*m and a nucleus with diameter 5 *μ*m. The cells and the well were uniformly filled with water.

The ^90^Y source was uniformly distributed throughout the well and between the cells (random locations outside of the cell volumes). Then, 10^10^ beta particles with energies randomly selected from the energy spectrum of ^90^Y were emitted from the source volume in random directions (see Fig. 3d). Finally, results were scaled to the total number of beta particles which corresponds to the cumulative activity in the well for 8 days irradiation. Additionally, the modelled cell culture was irradiated by simulation with a 6 MV FFF linac beam with similar SSD as the experimental set up. The spectrum of the FFF linac beam was obtained from the GATE software [19].

Moreover, additional simulations were performed to confirm that the ^90^Y-RNT experimental set-up; 1: results in a uniform dose distribution within the well, 2: the amount of cross–fire between adjacent wells in the same row does not affect the targeted dose, and 3: the well containing water can shield/attenuate the active rows from one another. Two adjacent wells were positioned in three rows; row 1 with active wells containing the highest ^90^YCl_3_ radioactivity, 288 kBq in 200 *μ*l cell culture medium in each well, row 2 with wells containing 200 *μ*l water, and row 3 with another two wells only containing 200 *μ*l cell culture medium in each well (see Fig. 3g).

#### Dose actor

The dose actor (a package within GATE) represents a cubic sensitive detector that can be voxelised into 3D rectangular or cubic voxels. Parameters such as dose, deposited energy, number of ionisations and number of beta particle hits were stored into this 3D matrix according to the spatial position of the hits for each individual simulated cell cytoplasm and nucleus. These parameters were calculated for varying ^90^Y activity concentrations used in the experiment. Additionally, the average number of hits per cell nucleus was calculated as a function of time for each ^90^Y activity to investigate the effect of initial dose rate. The number of beta particle hits per cell nucleus was calculated using

4 $$ N/\text{nucleus} = \frac{A_{n}}{\widetilde{A}} \widetilde{N}  $$

where *A*_*n*_ is the total number of decays within day *n* (where *n* = 1,2,3,...8) and $\widetilde {N}$ is the average number of beta particle hits to the nucleus corresponding to the total cumulative activity. For each $\widetilde {A}$, the corresponding $\widetilde {N}$ was determined from simulations.

### Cell survival study

For each of the EBRT, LDR and HDR–^90^Y cell irradiation experiments including their control cell plates, the MTS cell viability assay was added and cells were incubated at 37 ^∘^C for ≈ 3 h. Next, the viability of each cell culture was measured and *α* and *β* parameters were calculated from the linear quadratic (LQ) model [10]:

5 $$ \text{SF} = e^{-(\alpha D+\beta D^{2})}  $$

where SF is the cell survival fraction, *α* and *β* are the radiobiological parameters corresponding to the linear and nonlinear (quadratic) responses to the radiation dose. The *α* and *β* parameters were obtained by fitting the LQ model, Eq. 5, to the measured survival data. The SF was calculated for each cell line using Eq. 6 [25]:

6 $$ \text{SF} = \frac{\widetilde{OD}\text{in test wells} - \widetilde{OD} \text{in cell free wells}}{\widetilde{OD} \text{in control wells} - \widetilde{OD} \text{in cell free wells}}  $$

where $\widetilde {OD}$ is the mean optical density. The cell free wells contained the culture medium only.

The cells are incubated with ^90^YCl_3_ for 8 days; however, since the initial dose rate is exponentially decreasing over time, at a critical time (*T*_*crit*_) or dose rate (*R*_*crit*_) the DNA damage (the probability of causing DSB) effectively becomes insignificant [10]. For an exponentially decreasing dose rate, it is possible to calculate the critical time (*T*_*crit*_) after the start of treatment [10]. *T*_*crit*_ and *R*_*crit*_ for all the three lines are calculated for the highest initial dose rate using Eqs. 7 and 8 [10]:

7 $$ T_{crit} = - \frac{1}{\lambda} \text{ln} \bigg(\frac{0.693}{\alpha R_{0} T_{av}}\bigg)  $$

8 $$ R_{crit} = \frac{0.693}{\alpha\,T_{av}}  $$

where *λ*, *α*, *R*_0_ and *T*_*av*_ are the decay constant for ^90^Y, the radiobiological parameter of the cell line, the initial dose rate of ^90^Y RNT and the average doubling time of the cell line, respectively.

## Results

### Cell culture

**Fig. 4:**
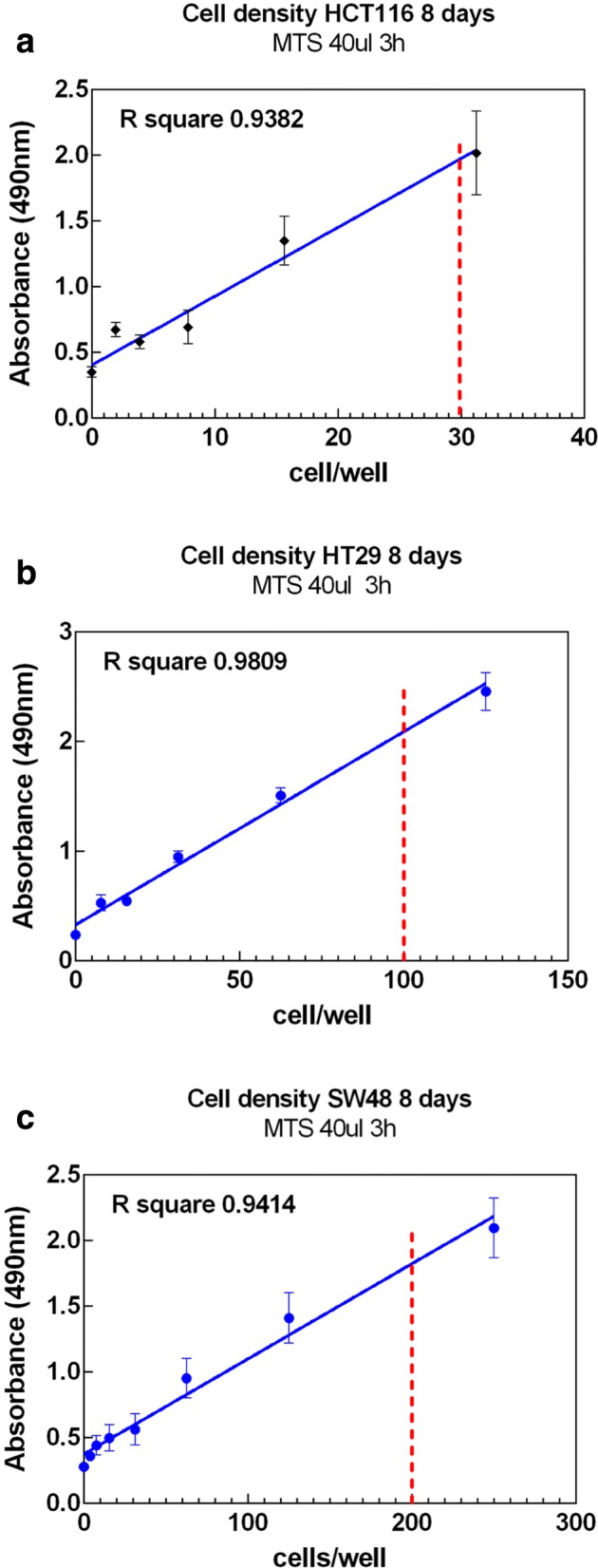
Linear regression fit for the cell density of three cell lines. The red dashed line indicates the number of cells per well used to culture the cells

Cell density measurements shown in Fig. 4 were used to find the optimal number of cells for both the EBRT and ^90^Y RNT experiments. The optimal cell densities in an exponential phase of growth for an 8 day density viability assay were calculated to be 30, 100 and 200 cells/well (in a 96-well plate) for HCT116, HT29 and SW48, respectively.

### Dosimetry

**Fig. 5:**
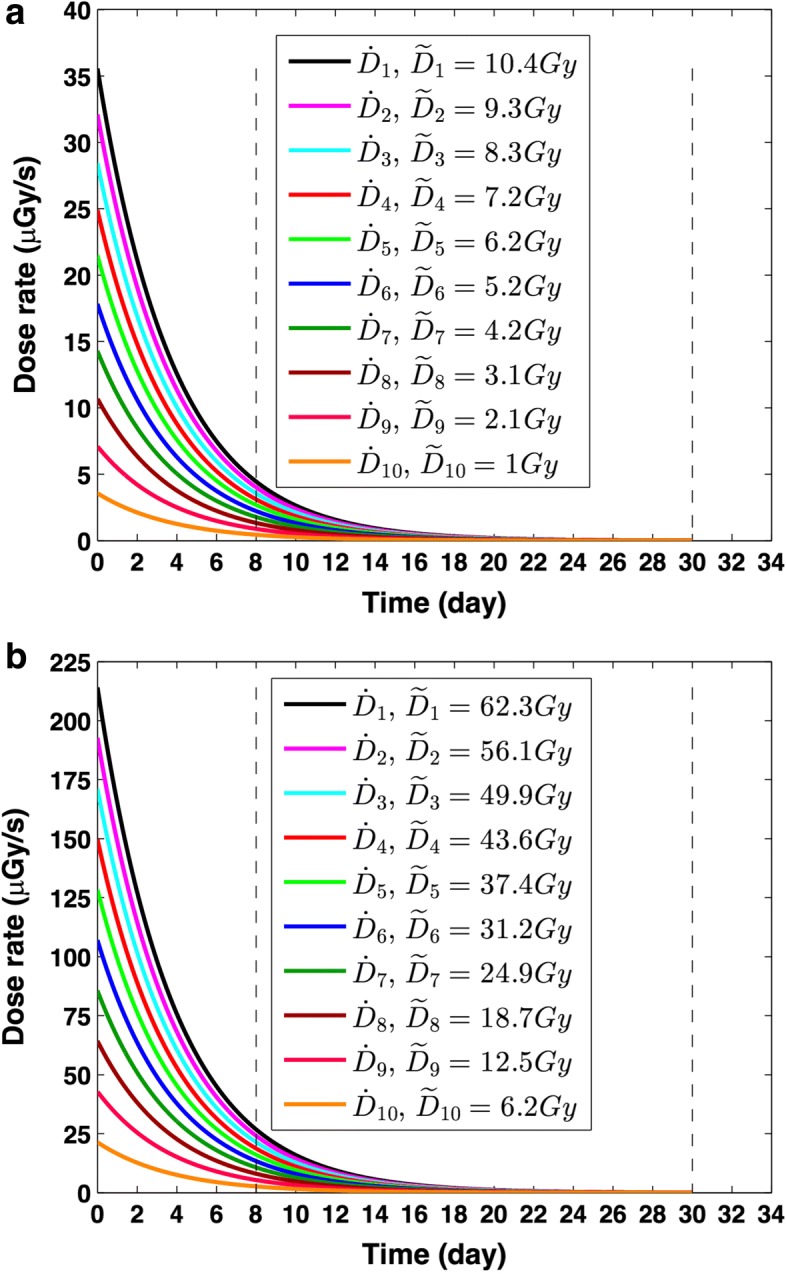
Dose rate ($\dot {D}$) and corresponding total dose (*D*) for different ^90^YCl_3_ concentrations calculated for *T* = 8 days: **a** LDR–^90^Y and **b** HDR–^90^Y.

A summary of total dose calculations and simulation results are presented in Tables 1 and 2. Figure 5 presents the calculated dose rates with corresponding total doses for both LDR and HDR–^90^Y. The activity for each delivered dose was selected to achieve the target total dose at *T* = 8 days incubation. In addition, a summary of total dose calculations performed by GATE, Matlab and OLINDA [21] is presented in Table 1. It is evident from Fig. 5 that different ^90^Y activities correspond to different initial dose rates, with a maximum ≈ 220 *μ*Gy/s, that decay exponentially over incubation time. In comparison, EBRT has a higher and constant dose rate, 277 Gy/h, (i.e. ≈ 10^5^ *μ*Gy/s) with shorter irradiation time, *T* ≈ 1.7 min.

**Table 1:** Summary of dose calculations for HDR and LDR–^90^Y activities

	*A*_*LDR*_ (kBq)	*A*_*HDR*_(kBq)	GATE (Gy)		Matlab (Gy)		OLINDA (Gy)	
	LDR	HDR	LDR	HDR	LDR	HDR	LDR	HDR
1	48.0	288	10.3 ± 0.1	61.7 ± 0.2	10.4 ± 0.2	62.3 ± 0.2	10.1 ± 0.1	60.6 ± 0.1
2	43.2	259	9.10 ± 0.1	55.5 ± 0.1	9.30 ± 0.1	56.1 ± 0.2	9.10 ± 0.1	54.5 ± 0.3
3	38.4	230	8.10 ± 0.1	49.4 ± 0.1	8.30 ± 0.1	49.9 ± 0.1	8.10 ± 0.2	48.5 ± 0.2
4	33.6	202	7.20 ± 0.2	43.2 ± 0.1	7.20 ± 0.1	43.6 ± 0.1	7.10 ± 0.1	42.4 ± 0.1
5	28.8	173	6.10 ± 0.1	37.0 ± 0.1	6.20 ± 0.2	37.4 ± 0.1	6.10 ± 0.2	36.6 ± 0.2
6	24.0	144	4.90 ± 0.1	30.9 ± 0.2	5.20 ± 0.1	31.2 ± 0.2	5.10 ± 0.2	30.1 ± 0.2
7	19.2	115	4.10 ± 0.2	24.7 ± 0.1	4.20 ± 0.2	24.9 ± 0.1	4.10 ± 0.2	24.2 ± 0.3
8	14.4	86.4	3.10 ± 0.1	18.5 ± 0.1	3.10 ± 0.1	18.7 ± 0.1	3.03 ± 0.1	18.2 ± 0.1
9	9.60	57.6	2.10 ± 0.1	12.3 ± 0.1	2.10 ± 0.1	12.5 ± 0.1	2.10 ± 0.2	12.1 ± 0.2
10	4.80	28.8	1.10 ± 0.1	6.20 ± 0.2	1.00 ± 0.1	6.20 ± 0.1	1.10 ± 0.3	6.10 ± 0.1

*A*_*LDR*_ and *A*_*HDR*_ represents the initial activity for the LDR– and HDR–^90^Y sources, respectively

**Table 2:** Summary of GATE simulation results for EBRT and ^90^Y irradiation

	EBRT			^90^Y		
	Cytoplasm	Nucleus	Cell	Cytoplasm	Nucleus	Cell
Irradiation time	< 1.7 min	< 1.7 min	< 1.7 min	8 days	8 days	8 days
Total no. of hits	3.11 × 10^10^	7.38 × 10^9^	3.85 × 10^10^	5.57 × 10^9^	9.97 × 10^8^	6.10 × 10^9^
$\widetilde {N}$	1.25 × 10^3^	295	1.540 × 10^3^	122	21	143
*D*_*int*_(Gy)	4.17 × 10^4^	8.28 × 10^3^	5.00 × 10^4^	4.26 × 10^4^	7.71 × 10^3^	5.03 × 10^4^
$\widetilde {D}$ (Gy)	10.5	10.1	10.3	10.4	10.1	10.3
Total no. of ionisations	1.49 × 10^10^	6.49 × 10^9^	2.14 × 10^10^	1.11 × 10^10^	2.78 × 10^9^	1.39 × 10^10^
Ave no. of ionisations	2.98 × 10^7^	1.30 × 10^6^	3.11 × 10^7^	2.23 × 10^7^	5.56 × 10^5^	2.29 × 10^7^

$\widetilde {N}$, *D*_*int*_ represent the average number of hits and integral dose respectively. The statistical uncertainties are negligible

### Cell survival

**Fig. 6:**
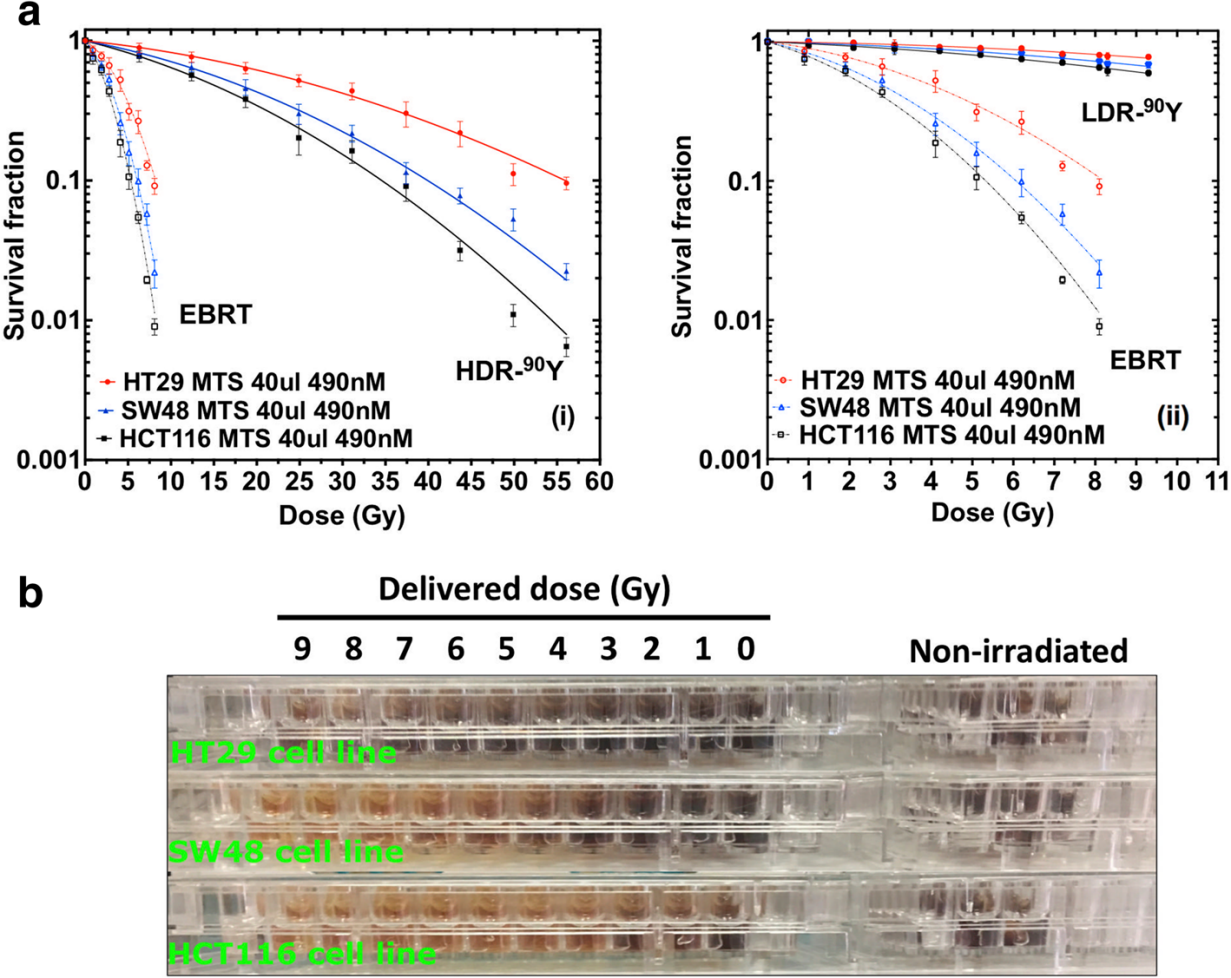
**a** Cell survival as a function of delivered dose for EBRT and (i) HDR–^90^Y RNT and (ii) LDR–^90^Y RNT, **b** MTS colorimetric dose response of EBRT at different delivered acute doses, darker brown colour represents higher number of viable cells in each well

Figure 6 presents the cell survival curves for all three cell lines for both EBRT and ^90^Y RNT. For each cell line, an EBRT dose > 8 Gy and a HDR–^90^Y dose > 56 Gy resulted in similar survival fractions and thus are not shown. Comparing the EBRT and HDR–^90^Y survival curves, similarities can be observed. Both EBRT and HDR–^90^Y resulted in an exponential dose response. However, at doses < 19 Gy, the HDR–^90^Y survival curve slope is shallower compared to that for EBRT. As the HDR–^90^Y dose rate decreases exponentially over the incubation time, for a given dose, HDR–^90^Y irradiation resulted in less cell kill than EBRT for all three cell lines. Figure 6a (ii) demonstrates the difference between cell survival for LDR–^90^Y RNT and EBRT, showing a negligible response from LDR–^90^Y RNT and resulting in negligible dose response compared to EBRT over the dose range studied, 1–8 Gy. The calculated *α*,*β* values are listed in Table 3. In comparison to EBRT, both of the *α* and *β* parameters for HDR–^90^Y are smaller. This is due to the relatively lower and exponentially decreasing dose rate of ^90^Y (i.e. maximum $\dot {D}_{0}\,\approx $ 0.77 Gy/h). Additionally, the *T*_*crit*_ for each cell lines is presented in Table 4. The *T*_*crit*_ for all three cell lines ranged approximately between 3 to 5.5 days. The HT29 cell line has the shortest *T*_*crit*_ (i.e. ≈ 3 days) which further confirmed that HT29 is the most radioresistant cell line.

**Table 3:** Summary of *α* and *β* parameters from EBRT and HDR–^90^Y irradiation

Cell lines	HCT116	SW48	HT29
*α*_*EBRT*_ (Gy ^−1^)	0.1981	0.1511	0.0842
*β*_*EBRT*_ (Gy ^−2^)	0.0439	0.0376	0.0239
$\phantom {\dot {i}\!}\alpha _{{}^{90}Y}$ (Gy ^−1^)	0.0350	0.0265	0.0145
$\phantom {\dot {i}\!}G \beta _{{}^{90}Y}$ (Gy ^−2^)	0.0009	0.0008	0.0005

**Table 4:** Critical times (*T*_*crit*_) and dose rates (*R*_*crit*_) for HDR–^90^Y RNT

Cell lines	*λ*(*h*^−1^)	*α*(*Gy*^−1^)	*R*_0_(*Gy*/*h*)	*T*_*av*_(*h*)	*T*_*crit*_(*days*)	*R*_*crit*_(*Gy*/*h*)
HCT116	0.0107	0.1981	0.792	20 [43]	5.883	0.1749
HT29	0.0107	0.0842	0.792	23 [44]	3.095	0.3578
SW48	0.0107	0.1511	0.792	24 [43]	5.538	0.1911

*λ*, *α*, *R*_0_ and *T*_*av*_ are the decay constant for ^90^Y, the radiobiological parameter of the cell line, the initial dose rate of ^90^Y RNT and the average doubling time of the cell line, respectively

Figure 6b presents the colorimetric dose response of EBRT at different delivered acute doses (e.g. 1–9 Gy). This figure shows all three cell lines with added MTS solution and incubated for 3 h. The dark brown colour represents a higher number of viable cells in each well. The HT29 cell line is shown to be the most radioresistant cell line. This is evident from Fig. 6b as more cells survived at higher dose (darker colour). The HCT116 cell line was the most radiosensitive cell line showing less cell survival at even a low delivered doses (less dark). The *α*/*β* ratios were determined from the LQ model (Eq. 5) for both EBRT and HDR–^90^Y. For LDR–^90^Y, the percentage of cell kill was insignificant to obtain meaningful *α*/*β* values. The alpha values derived from the survival curve were 0.01 ± 0.0029, 0.007 ± 0.0017, and 0.0023 ± 0.001 Gy ^−1^ for HCT116, SW48, and HT29 cell lines, respectively.

### Monte Carlo simulation

**Fig. 7:**
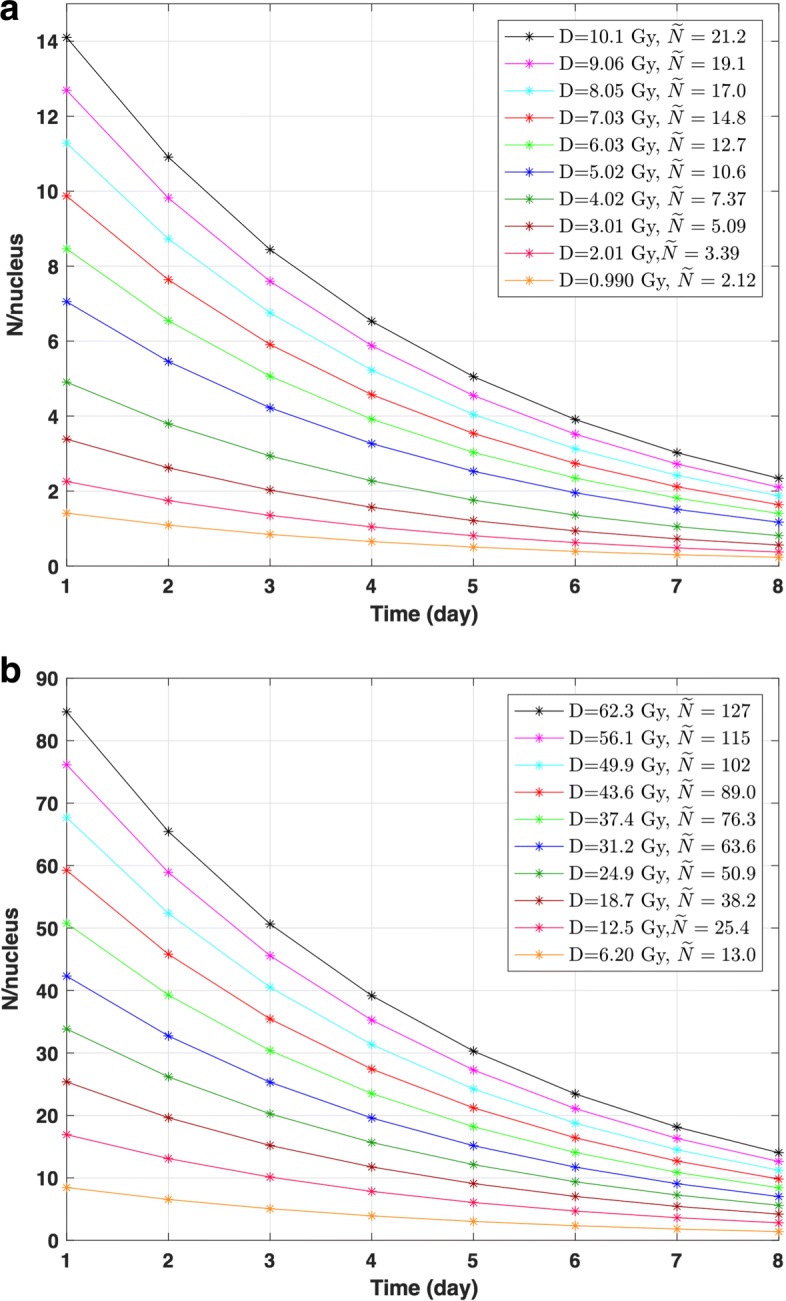
Histograms of the average number of beta particle hits per cell nucleus *N*/nucleus binned into each day of irradiation and corresponding average number of hits $\widetilde {N}$ for the total 8-day irradiation time calculated from simulations for **a** LDR–^90^Y and **b** HDR–^90^Y. *D* is the total dose (*D*)

Figure 7 presents histograms of the number of beta particle hits per cell nucleus during the 8-day irradiation time for both LDR– and HDR–^90^Y calculated by the simulations. The number of hits per cell nucleus decreases as the activity decays with time. The mean separation between energy deposition events at an LET value of ≈ 1 keV/ *μ*m (i.e. LET of beta particles emitted from ^90^Y) is larger than the DNA double helix’s diameter (i.e. ≈ 2 nm)[26]. Therefore, for a single-beta particle hit, the probability of a DSB occurring is lower for betas with LET above the mean compare to below the mean [26]. Thus, the minimum number of hits needed to give a nonzero probability of DSB is ≈ 2. It is evident for LDR–^90^Y that the average number of hits per cell nucleus is ≲ 2 for doses ≲ 3 Gy during the 8 days irradiation time. Although for doses $\gtrsim $ 3 Gy the average number of hits per cell nucleus is $\gtrsim $ 2, it decreases rapidly and falls below 2 after ≈ 5 days of irradiation for doses ≲ 5 Gy. For higher doses, *N*/nucleus is ≲ 2 after ≈ 7–8 days. For HDR–^90^Y, similarly, the number of hits per cell nucleus decreases during the 8 days of irradiation time but the average number of hits per cell nucleus remains above 2 for all ^90^Y doses investigated. For EBRT, $\widetilde {N}\,\approx $ 295 and 30 for 10 and 1 Gy total dose, respectively.

**Fig. 8:**
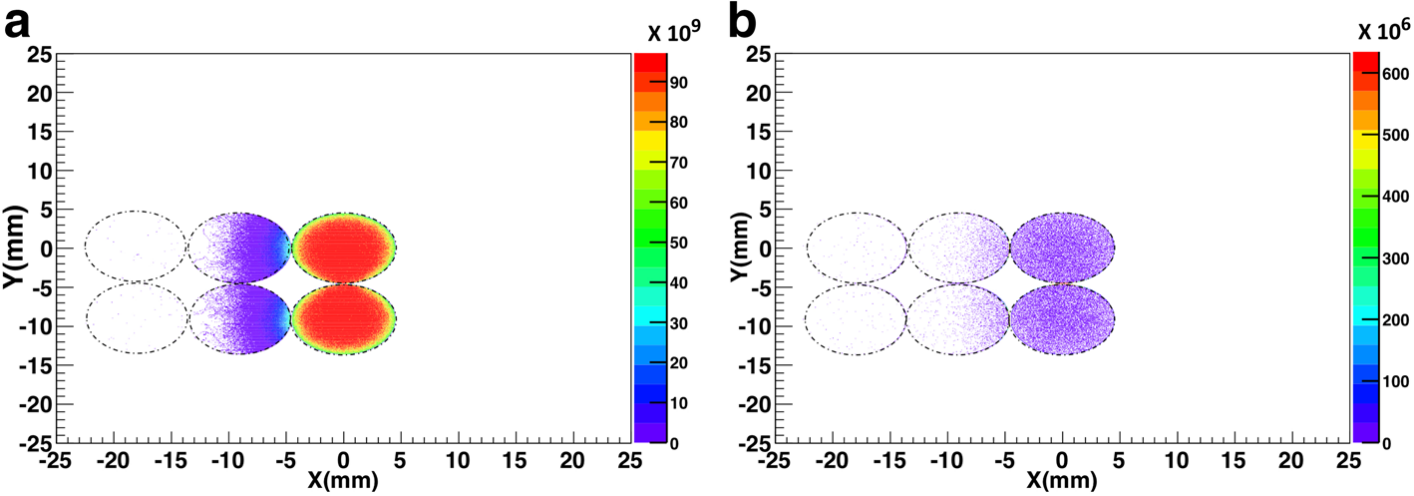
**a** Two-dimensional energy histogram for six adjacent wells by integrating the corresponding 3D energy distribution along the *Z* direction. The scale bar is in MeV. **b** Two-dimensional beta particle fluence histogram for six adjacent wells by integrating the corresponding 3D fluence distribution along the *Z* direction. Black dotted circles indicate the edges of the wells

Furthermore, Fig. 8a demonstrates a two-dimensional (2D) histogram of the energy distribution in all six wells. As it is evident from Fig. 8a, most of the voxels in each active well receive a similar energy deposition (the red region). Figure 8b shows a 2D histogram of beta particle fluence for all six wells. The beta particle fluence histogram represents the number of particles that entered into each well from outside (the amount of cross-fire between adjacent wells). Figure 8b demonstrates a beta particle equilibrium between the adjacent active cell wells. Each active well in row 1 equally received 6% from the adjacent well (the purple region). Additionally the cross-fire between the adjacent wells did not change the total energy deposition in each well (each well received approximately 61.7 Gy).

## Discussion

In comparison to the established clonogenic assay, the MTS (or MTT) assays are sensitive and accurate methods with several advantages such as requiring a short time to assess the viability, acquiring the results easily and accurately [25, 27–32]. It measures all viable cells thus representing cells from a true tumour population rather than just clonogenic cells (i.e. cells that can form colonies) [33, 34].

Previous studies have shown that the cells exposed to the higher radiation doses cannot regain their exponential growth in a single MTT or MTS assay, and multiple MTS assays are required to obtain measurements of radiation survival that is comparable to that of clonogenic assays [27, 29]. Although previous studies have shown that MTS and MTT assays are suitable to investigate the radiation dose-response analysis [29], the clonogenic assay has been preferred to measure survival after radiation exposure, as it measures the sum of all modes of cell death, encompassing both early and late events such as delayed growth arrest [35]. Therefore, the *α*/*β* ratios derived from a metabolic activity assay at a single time point should be used cautiously in further radiobiological modelling. Additionally, the cells sustaining radiation-induced damage may exhibit delayed effects and there could be a lag between irradiation and subsequent biological events (e.g. cell cycle arrest, caspase activation) leading to cell death [10, 36]. The acute DNA damage induced by radiation and the subsequent cellular responses are influenced by multiple factors including radiation characteristic, dose rate and cell cycle phase [10, 36]. In RNT, the dose rate is one of the most important factors influencing the cell damage, cell repair and also the effective treatment time. The *T*_*crit*_ for all three lines ranges approximately 3–5.5 days. In this study, the MTS assays were performed eight days after the initial addition of ^90^YCl_3_, on average ≈ 4 days beyond these critical times where the treatment is effectively being insignificant and the delayed effects could occur. Thus, performing the MTS assay after the actual treatment time could still include the cell death due to the delayed effects.

The cell survival results provide insight into different damage responses to EBRT and ^90^Y RNT. With EBRT, the dose was delivered at a constant dose rate of 277 Gy/h in ≈ 1.7 min, whereas ^90^Y was delivered at a lower dose and varying rate (i.e. 0.013–0.13 Gy/h and 0.077–0.77 Gy/h) over 8 days. The dose rate and nature of radiation clearly affects the nature of cell damage. There are two types of cell damages: (1) double-strand break (DSB), where two proximal DNA strands are broken simultaneously by a single radiation event causing lethal damage (LD) (i.e. cell death) and (2) single-strand break (SSB), where each DNA strand broken by independent radiation events. When only a single DNA strand is damaged, the cell is considered sublethally damaged (SLD) and has the ability to repair itself typically within in 0.5-3 hours [37]. Therefore, at lower dose rates, the probability of accruing two sequential hits to damage before DNA repair is less. Moreover for an exponentially decreasing dose rate, the probability of DSB is even lower. Therefore LDR–^90^Y RNT is less effective at causing lethal damage than HDR–^90^Y RNT and EBRT.

The rate of cell kill through the DSB process is measured from the response component of the survival curve parameterised by *α*. The rate of cell kill through the SSB process is measured from the quadratic part of the survival curve, parameterised by *β* which is highly dependent on the initial dose rate. The value of *α* depends on the the radiation type. For high LET radiation such as alpha particles, DSB dominates the cell kill process and therefore *α* has higher significance than *β*. Therefore, for a low and exponentially decreasing dose rate ^90^Y source, there is less contribution to SF from the *βD*^2^ component than from the *α* term. This is evident from Fig. 6a (i and ii), as SSB repair results in the formation of a shoulder for the HDR–^90^Y survival curve. However, as the EBRT dose rate is significantly higher than HDR–^90^Y RNT (by a factor 400), the rate of lethal SSB production is much higher. Consequently, this results in delivering more lethal damage to cells at an even lower EBRT dose range (compared to ^90^Y doses). Moreover for LDR–^90^Y (0.013-0.13 Gy/h), there is even less contribution from the *βD*^2^ component since the rate of SSB repair occurs even more rapidly (see Fig. 6a (ii)) than the rate of damage. Simulation results further confirmed that for both of HDR– and LDR–^90^Y, for smaller delivered activities and doses (i.e. ≲ 3 Gy) the average number of hits per cell nucleus is ≲2. Additionally for doses $\gtrsim $ 3 Gy, although the average number of hits is $\gtrsim $ 2, the number of beta particle hits per cell nucleus decreases and falls below 2 after ≈ 5 days of irradiation. This is also consistent with the calculated *T*_*crit*_ for each cell lines (i.e. 3–5.5 days). This suggests that for LDR– ^90^*Y* only ≈ 1–2 half-lives of delivered dose can potentially cause direct cell nucleus DNA damage.

All three cell lines showed higher sensitivity to EBRT than ^90^Y. The HT29 and HCT116 cell lines were the most radioresistant and radiosensitive cell lines, respectively. This is also consistent with previous studies [38, 39]. Similar sensitivity for both cell lines was observed for ^90^Y irradiation results. The *α*/*β* ratios for both EBRT and ^90^Y were derived from Eq. 5. However, several models have been developed to incorporate the effect of DNA repair and the exponentially changing dose rate of RNT [10, 37]. The extended LQ model includes the kinetics of DSB creation, repair, and misrepair to estimate the true fraction of surviving cells in an irradiated cell population (Eqs. 9a and 9b).

9a $$\begin{array}{*{20}l} \text{-ln(SF)} &= \alpha\,D+\beta\,G\,D^{2}  \end{array} $$

9b $$\begin{array}{*{20}l} G &= \frac{2}{D^{2}}\,\int_{-\infty}^{\infty}\,\dot{D}(t)\,dt\,\int_{-\infty}^{t}\,\dot{D'}(t')\,\,e^{- \mu\,(t-t')}\,dt'  \end{array} $$

where *G* is the Lea–Catcheside factor and *μ* is the DNA repair time constant [40]. The first integral represents the physical absorbed dose. The integrand of the second integral over *t*^′^ refers to the first SSB of two DSBs required to cause lethal damage. Also, the integral over *t* refers to the second SSB of remaining of two DSBs to cause the unrepairable lethal damage. The exponential term describes the repair and reduction in 2 SSB → DSB process due to decreasing dose rate. For a constant EBRT dose rate where the dose is delivered acutely and the irradiation time is very short, the *G*–factor approaches unity and Eq. 9a simplifies to its general form (Eq. 5). This means the rate of SSB → DSB process production is higher than the DNA repair rate, and therefore, the kinetics of DSB creation are negligible.

In comparison to EBRT, the ^90^Y RNT dose rate is much lower and exponentially decreasing (Eq. 1), so the difference in the kinetics of DSB creation has more impact on cell survival. The *G* factor can be derived for ^90^Y radiation using Eqs. 10a, 10b.

10a $$\begin{array}{@{}rcl@{}} G\,=2\,\bigg(\frac{\dot{D_{0}}}{D}\bigg)^{2}\,\int_{0}^{T} e^{-\lambda t}\,dt\,\int_{-\infty}^{t} e^{-\lambda t'}\,e^{- \mu\,\left(t-t'\right)}\,dt'  \end{array} $$

10b $$\begin{array}{@{}rcl@{}} = \frac{2}{\lambda-\mu}\,\bigg(\frac{\lambda}{1-e^{-\lambda T}}\bigg)^{2}\,\bigg(\frac{1-e^{-(\lambda+\mu) T}}{\lambda+\mu}\,-\frac{1-e^{-2 \lambda T}}{2\lambda}\bigg)  \end{array} $$

For long irradiation times (i.e. *T* → *∞*) the *G* factor reduces to:

11 $$\begin{array}{@{}rcl@{}} G_{\infty}\,=\frac{\lambda}{\lambda+\mu}\,=\frac{\tau_{1/2}}{T_{1/2}+\tau_{1/2}}  \end{array} $$

where *T* and *τ* are the physical half-life of ^90^Y and DNA repair half-life, respectively. Since *G*_*∞*_ only depends on the physical half-life of ^90^Y and the DNA repair half-life, its value for both HDR– and LDR–^90^Y RNT can be determined using Eq. 11. However, as the DNA repair half-lives for all the three cell lines in our study were not experimentally derived, *G*_*∞*_ for HDR– and LDR–^90^Y was calculated using Eq. 11 and assuming an average value of *τ*_1/2_=1.5 h [11], giving *G*_*∞*_=0.022. Such a relatively small *G*_*∞*_ value for ^90^Y suggests less contribution from 2 SSB → DSB production (e.g. *β* *D*^2^) as the dose rate is relatively lower compared to EBRT. In comparison, *G*_*∞*_≈1 for EBRT, using an irradiation time ≈ 1.7 min. This is more evident for the LDR–^90^Y survival curve since for a given dose, the initial dose rate is ≈ 0.013–0.13 Gy/h. This is also consistent with the simulation results. For example, for LDR–^90^Y, for smaller delivered activities and doses (i.e. ≲ 3 Gy), the average number of hits per cell nucleus is ≲ 2 during the 8 days irradiation time (see Fig. 7a). Therefore, there will be very low probability for 2 SSB → DSB process at this dose rate levels. These results highlight the importance of initial dose rate in radionuclide therapy.

In RNT, dose rate is inversely proportional to the radionuclide half-life for a given total dose. Therefore, a longer half-life radionuclide such as ^90^Y delivers dose at a relatively lower rate for a fixed total dose. Equation 11 implies that for a similar absorbed dose, radionuclides with a shorter half-life could potentially result in better dose response for RNT. Since radionuclides with shorter half-lives deliver dose at much higher initial rate, the DNA damage process can compete with DNA repair rate and thus result in better dose response.

**Fig. 9:**
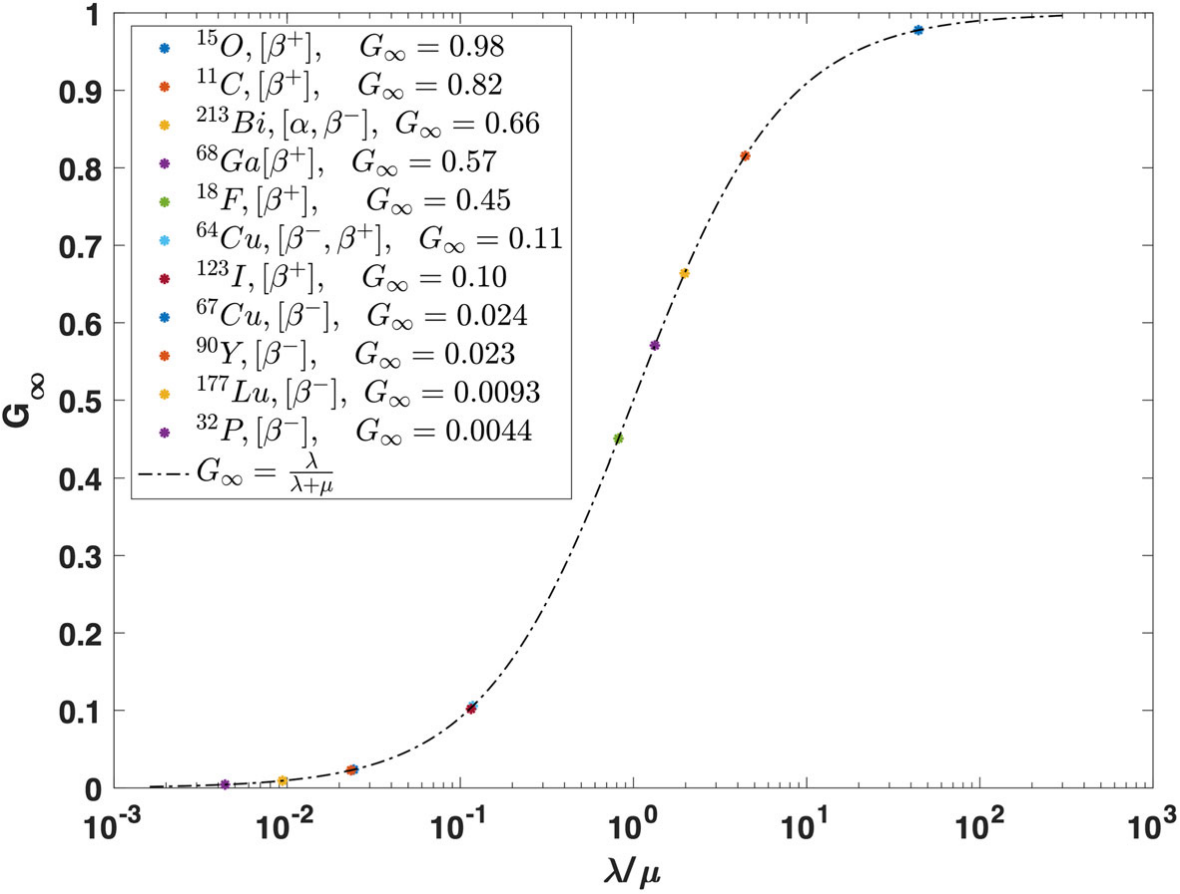
*G*_*∞*_ for common medical isotopes

The *G*_*∞*_ factor was calculated and plotted (see Fig. 9 in Appendix) for a few commonly used isotopes in RNT and emission tomography for comparison. These calculations assume *μ* to be 0.42 h ^−1^ (ln(0.5)/15 hours). The *G*_*∞*_ factor also plays an important role for high LET radiation. For example ^213^Bi with a 46 min half-life which decays ≈2*%* as high LET alpha and ≈98*%* low energy beta particles, delivers dose at a reasonably high dose rate. Currently ^213^Bi is the only radionuclide that can deliver dose at a dose rate close to the EBRT dose rate (*G*_*EBRT*_ ≈ 1, *G*_*Bi*_ ≈ 0.7). The ability to deliver a very localised dose at very high dose rate has a significant impact on the RNT [41]. For both EBRT and ^90^Y, the HT29 and HCT116 cell lines were the most radioresistance and radiosensitive cell lines, respectively. HT-29 cells have the TP53 mutation which may increase the cell resistance to radiation [38]. The *α*/*β* derived from the HDR–^90^Y cell survival curve was larger than that derived from EBRT by a factor of ≈ 8 for all three cell lines. This can be attributed to a relatively lower dose rate of ^90^Y. Also for each given dose, the total HDR–^90^Y absorbed dose (6.2–55.5 Gy) was up to six times greater than the EBRT dose (1–9 Gy).

Additionally, different *α* and *β* values derived for HDR–^90^Y RNT and EBRT reflect on the different relative radiobiological effectiveness of ^90^Y RNT compared to EBRT.

These results are subject to several assumptions. Firstly, for all the calculations and formalisms, it was assumed that there is no significant cell repopulation during 8 days of incubation. A more complex formulation is required to offset the repopulation effect [10, 42]. Secondly, it was assumed that ^90^YCl_3_ was uniformly distributed in the well. This assumption was used to calculate the absorbed dose to cells for each ^90^YCl_3_ activity.

## Conclusions

We benchmarked the effectiveness of EBRT to ^90^Y RNT in vitro specific to CRC cell lines. For the HT29 cell line, we found *α*/*β* ≈ 31 for HDR–^90^Y compared to ≈ 3.5 for EBRT. For LDR–^90^Y, we found insignificant cell death at doses < 10 Gy compared to EBRT. Simulation results also demonstrated that for ^90^Y doses ≲ 3 Gy, the average number of hits per cell nucleus ≲ 2 indicating insufficient delivered lethal dose. Similarly, for ^90^Y doses $\gtrsim $ 3 Gy the average number of hits per cell nucleus falls below ≈ 2 after ≈ 5 days of incubation time. Our simulation results also demonstrated that the average number of hits per cell nucleus for EBRT is ≈ 14 times larger than ^90^Y RNT. Therefore, our results demonstrate that LDR–^90^Y is radiobiologically less effective than EBRT. However, HDR–^90^Y at ≈ 56 Gy was shown to be radiobiologically as effective as acute ≈ 8 Gy EBRT. These results demonstrate that the efficacy of RNT is dependent on the initial rate at which dose is delivered. The RNT dose rate is inversely proportional to the radionuclide half-life for a given total dose. Therefore, a longer half-life radionuclide such as ^90^Y requires a higher initial activity to attain higher initial dose rate, and a larger total dose in Gy delivered is required to achieve a treatment outcome that is equally as effective as EBRT.

## Appendix
